# Modified In-Office Superior Laryngeal Nerve Steroid Injection Technique for the Treatment of Chronic Cough

**DOI:** 10.3390/jcm15134883

**Published:** 2026-06-23

**Authors:** James Tsimiklis, Theodore Athanasiadis

**Affiliations:** 1 Department of Otorhinolaryngology—Head & Neck Surgery, Flinders Medical Centre, Adelaide 5042, Australia; 2 Department of Otorhinolaryngology—Head & Neck Surgery, Flinders University, Adelaide 5042, Australia

**Keywords:** chronic cough, superior laryngeal nerve, modified technique, steroid injection, in-office

## Abstract

**Objectives:** To compare an endoscopically guided, modified in-office internal superior laryngeal nerve (iSLN) percutaneous steroid injection technique with a traditional landmark-guided percutaneous approach for refractory chronic cough. **Methods:** Single-centre retrospective comparative cohort study of those with chronic cough >8 weeks adjudicated as neurogenic/hypersensitivity-related after structured evaluation and management of common aetiologies. Consecutive patients treated at a tertiary laryngology service from January 2021 to January 2025 were identified. Patients underwent either landmark-guided percutaneous iSLN block (unmodified) or iSLN percutaneous block under flexible nasolaryngoscopic visualisation (modified), enabling real-time confirmation of needle position and routine bilateral treatment with partial superficial laryngeal mucosal instillation. Primary outcome was patient-reported improvement (Y/N; 1–10 severity scale). Secondary outcomes included Leicester Cough Questionnaire, Cough Severity Index, Newcastle Laryngeal Hypersensitivity Questionnaire, Reflux Severity Index, Voice Handicap Index-10, repeat procedures, and adverse events. **Results:** Of 142 patients (median age 62.8 years; 75% female), 65 underwent landmark-guided injection and 77 underwent the modified endoscopically guided technique. At most recent follow-up, global improvement was reported by 84.4% (65/77) in the modified cohort versus 47.7% (31/65) in the unmodified cohort. Median symptom reduction was greater with the modified approach (77.8% (IQR 61.3–86.6)) than among unmodified responders (50.3% (IQR 25.0–75.0)). Across all validated patient-reported outcome measures, the modified technique demonstrated more pronounced improvement than the landmark-guided approach. Minor adverse events were uncommon (modified = 6.5%, unmodified = 4.6%). **Conclusions:** Endoscopically guided modified iSLN steroid injection with routine bilateral targeting is associated with greater patient-reported improvement and superior validated cough outcomes than landmark-guided injection, without added significant risk.

## 1. Introduction

Cough is a fundamental airway defence mechanism and the most common symptom encountered in primary care [1]. When intact and effective, it serves to protect lung health by preventing aspiration and enhancing mucociliary clearance through a coordinated three-phase motor pattern that generates high expiratory airflow to mobilise and expel secretions and debris [2]. However, when cough becomes chronic or hypersensitive, it shifts from protective to pathological. It can prove to hold a great burden against those in which it persists from a quality-of-life perspective, leading to patient distress and substantial healthcare utilisation [3].

Chronic cough denotes a cough lasting for a greater period than 8 weeks [4]. This condition has a global prevalence of 7.6–11.7% [5]. In many patients, cough can persist despite treatment of traditional precipitating factors such as asthma, gastro-oesophageal reflux disease (GORD), sinonasal disease, lower respiratory tract disease (LRTD), or medication-related stimulation [6]. Neurogenic chronic cough is thought to result from laryngeal sensory neuropathy with pathological plasticity of the vagally mediated cough reflex arc. Peripheral afferent fibres in the laryngeal and proximal airway mucosa become hyperresponsive to normally innocuous stimuli, and central processing within the brainstem cough network in the region of nucleus tractus solitarius amplifies and sustains cough output despite limited ongoing physiological benefit. This maladaptive upregulation is triggered following an initiating insult, commonly infection or inflammation, and altered receptor expression and excitability can lower activation thresholds and increase firing. This produces a persistent irritable-larynx phenotype [2].

Management of refractory chronic cough involves non-pharmacological therapy such as cough suppression techniques and laryngeal retraining therapy, typically facilitated by a speech and language pathologist. Pharmacological techniques employ neuromodulators such as low-dose gabapentinoids, amitriptyline, or opiates that can reduce cough frequency in some patients. These medications often have side-effects, risk of tolerance, and variable adherence [6]. Injectable agents such as botulinum toxin and synthetic corticosteroids are increasingly used as minimally invasive, office-based adjuncts for patients with refractory symptoms. These aim to modulate the dysfunctional laryngeal component of cough hypersensitivity when behavioural therapy and neuromodulators are insufficient or poorly tolerated. Laryngeal botulinum toxin A has been proposed to act via a dual mechanism, weakening laryngeal adductor muscles through inhibition of acetylcholine release and potentially attenuating sensory neurotransmission via the substance-P-mediated pathways. EMG-guided bilateral thyroarytenoid injections in carefully selected recalcitrant cases have been associated with clinically significant improvements in patient-reported outcomes, including reduced cough severity and improved cough-related quality of life [7]. Injectables have also been used to target the internal branch of the superior laryngeal nerve (iSLN), a peripheral afferent driver of laryngeal hypersensitivity. The earliest contemporary clinical series describing this approach introduced in-office percutaneous iSLN blockade at the thyrohyoid membrane using a 1:1 mixture of particulate corticosteroid and local anaesthetic, with the intent of reducing aberrant sensory input to the central cough network. This treatment was associated with important reductions in cough severity scores and established iSLN steroid blockade as a procedural adjunct alongside contemporary therapies [8].

Despite demonstrated efficacy, the traditional landmark-guided iSLN injection technique holds various limitations. It typically targets a unilateral side, many patients require numerous repeat injections in subsequent visits if cough persists, and there is concern that incautiously conducted bilateral blocks utilising this method could temporarily reduce protective laryngeal sensation and precipitate aspiration. Moreover, delivering the injectate precisely to the vicinity of the iSLN can be technically challenging utilising external landmarks alone, particularly in patients with challenging neck anatomy or an absence of unilateral trigger point. A misplaced injection may reduce efficacy and deplete the utility of the intervention [9].

To address these issues, we developed a modified in-office iSLN block technique employing flexible nasolaryngoscopy to directly visualise the larynx during the injection. Via endoscopic observation, the surgeon can confirm percutaneous needle placement and perform bilateral injections into the region of the iSLN as well as the laryngeal superficial mucosa. This study investigates this modified approach, assessing if it produces improved clinical outcomes compared to the landmark-guided technique.

## 2. Methods

### 2.1. Study Design

This retrospective audit was conducted as a quality assurance project (Register ID: 4972) and was granted ethics exemption by the Southern Adelaide Clinical Human Research Ethics Committee. In line with the National Safety and Quality Health Service Standards and the SALHN Four Fields of Enquiry continuous improvement framework, quality assurance and service evaluations do not require formal ethics approval. All data were de-identified and handled in accordance with institutional privacy protocols. It was prepared in accordance with the Strengthening the Reporting of Observational Studies in Epidemiology (STROBE) reporting guideline.

Clinical records from January 2021 through to January 2025 were reviewed to identify patients with chronic cough who underwent in-office iSLN block injection at our tertiary centre (Adelaide & Hills ENT, South Australia, Australia). All procedures were performed by a single surgeon. Patients were characterised based on the injection technique used: the unmodified group (landmark-guided percutaneous block) and the modified group (endoscopically guided percutaneous block).

### 2.2. Patient Population

Recruitment to the study was conducted in a structured approach to systematically identify all patients that received an iSLN block for chronic cough. Clinical records of all adult patients (age ≥ 18) with a chronic cough lasting >8 weeks that was unexplained by alternative causes after workup and treatment of common aetiologies were included. iSLN block was offered only after structured evaluation and management of recognised precipitants of chronic cough—including GORD, allergic and sinonasal disease, asthma, and medication-related cough—had been addressed or excluded. In the vast majority of cases, patients had also undergone a trial of speech–language pathology (SLP) cough suppression therapy and at least one neuromodulator agent prior to referral for procedural intervention, consistent with contemporary cough management guidelines. No patients were excluded on the basis of anatomical factors alone; however, cases where neck anatomy substantially precluded reliable external landmark identification were preferentially managed with the modified endoscopic technique, which may represent a minor source of selection bias. Medical comorbidities such as allergic processes, asthma, reflux, smoking or vaping and active pulmonary disease were noted. This tertiary laryngology clinic transitioned from the standard iSLN injection technique to using the modified technique in February 2024. Records which had incomplete follow-up data or included cough due to other untreated pathology such as pulmonary malignancy were excluded.

### 2.3. Outcome Measures

The primary outcome was patient-reported improvement in cough symptoms post-injection, captured at the most recent follow-up visit. Patients were first asked to provide dichotomous responses relating to global improvement relative to pre-injection baseline (Y/N). They were then asked to assess on a 1–10 scale (1 = complete resolution; 10 = worst possible cough) the severity of their cough. A clinically significant response was defined a priori as ≥50% improvement, consistent with symptomatic and quality-of-life benefit. Complete or near-complete resolution was defined as ≥90%.

Secondary outcomes included changes in validated patient-reported outcome measures: the Leicester Cough Questionnaire (LCQ), Cough Severity Index (CSI), Newcastle Laryngeal Hypersensitivity Questionnaire (NLHQ), Reflux Symptom Index (RSI), and Voice Handicap Index-10 (VHI-10). These instruments were administered at baseline and follow-up, and change scores were calculated as post-injection minus pre-injection values. Additional secondary endpoints included whether repeat injection was pursued and laterality of injections as an indicator of treatment adequacy. All procedural adverse events or complications were noted.

### 2.4. Statistical Analysis

Continuous data are presented as mean and standard deviation or median and interquartile range (IQR), as appropriate. Categorical variables were summarised as counts and percentages. Baseline characteristics between the unmodified and modified groups were compared using Mann–Whitney U tests for continuous variables and Fisher’s exact tests for categorical variables. Within-group pre- to post-injection changes in patient-reported outcome measures were assessed using Wilcoxon signed-rank tests.

### 2.5. Injection Technique

Both groups underwent laryngostroboscopy at each clinical visit and prior to intervention.

#### 2.5.1. Unmodified

The cricothyroid space and hyoid landmarks were identified via palpation. The site of injection was chosen based on patient history of laterality and clinical trigger point examination. This involved the application of gentle pressure over a unilateral thyrohyoid space to determine if it consistently provoked cough. In the event of no evident lateralisation on history or examination, bilateral injection was employed. After preparing the neck with antiseptic, a 27-gauge needle was inserted percutaneously at the lateral thyrohyoid membrane, inferior to the greater cornu of the hyoid, in the region of the internal superior laryngeal nerve. The surgeon aspirated to ensure avoidance of vascular entry, then injected 1 mL of naropin 1% (ropivacaine) and 1 mL of kenacort 40 mg (triamcinolone acetonide) per side in close proximity to the iSLN. This formulation and volume were standardised across all procedures in both cohorts. Patients were observed for 20 min following completion of the procedure for the development of any early adverse events.

#### 2.5.2. Modified

For the modified endoscopically assisted percutaneous technique, a flexible nasolaryngoscope was introduced transnasally after the application of topical anaesthetic spray (Co-phenylcaine Forte; lidocaine and phenylephrine; Aspen Pharmacare, St Leonards, NSW, Australia) to the nasal passage. The larynx was visualised and remained in view throughout the procedure. Under endoscopic view, the same percutaneous approach was used, but the surgeon was able to watch the needle tip tenting the mucosa in the region of the aryepiglottic fold and false fold. Approximately half the injectate was instilled in the region of the iSLN via anatomical landmarks and the remainder was instilled into the superficial laryngeal mucosa of the aryepiglottic and false folds. The injectate formulation was identical to the unmodified technique (1 mL ropivacaine 1% and 1 mL triamcinolone acetonide 40 mg per side), administered bilaterally as the procedural default. The modified technique requires a trained assistant to hold the flexible nasolaryngoscope, allowing the operating surgeon to perform the percutaneous injection under continuous endoscopic visualisation. After receiving the modified bilateral block, patients were similarly briefly observed.

## 3. Results

### 3.1. Cohort Characteristics

A total of 142 patients (median age 62.8 years, range 24–89, 75% female) were identified, consisting of 65 in the unmodified iSLN injection group (median age 62 years (IQR 48–73); female 49/65, 75.4%) and 77 in the modified technique group (median age 67 years (IQR 55–75); female 57/77, 74.0%). No patients were lost to follow-up. In both cohorts, chronic cough duration prior to intervention exceeded 8 years (mean; unmodified = 10.0, modified = 8.8 years). There was no statistically significant between-group difference in cough duration (Mann–Whitney U test, *p* = 0.34). There were no significant between-group differences in age (Mann–Whitney U test, *p* = 0.201) and sex (Fisher’s exact test; *p* = 1.000) distribution. The modified group had a higher prevalence of asthma (33/77, 42.9%) compared to the unmodified group (17/65, 26.2%; *p* = 0.03). A greater proportion of the modified group also had documented gastro-oesophageal reflux disease (54/77, 70.1%) versus the unmodified group (35/65, 53.8%; *p* = 0.05). The incidence of allergic rhinitis (26% vs. 15%; *p* = 0.12) and post-nasal drip complaints (39% vs. 26%; *p* = 0.11) was higher in the modified cohort, though not reaching statistical significance. There were a limited number of patients with lower respiratory tract disease, amounting to 14% in the modified cohort versus 4.6% in the unmodified cohort. Only one patient, a member of the modified group, had a history of vagal nerve injury (see Table 1).

### 3.2. Prior Cough Treatments

Patients typically had undergone extensive therapies before consideration of iSLN block, with refractory cough persisting despite multiple interventions. In the unmodified group, 81.5% had participated in cough suppression therapy with a speech and language pathologist, compared to 81.8% in the modified group (Fisher’s exact test *p* > 0.99). GORD management had been attempted by 86% of study participants, inhaled asthma therapy by 80%, and nasal steroid sprays for allergic rhinitis by 60%. Neuromodulator medications had been trialled by 49% and 58% of the unmodified and modified cohorts respectively. Notably, 16 patients (24.6%) in the unmodified group and 12 patients (15.6%) in the modified group had undergone prior ineffective laryngeal botulinum toxin injections for cough before resorting to iSLN block. A limited number of participants had tried empiric treatments such as oral steroid tapers, morphine or codeine cough suppressants, or respiratory biologic agents without lasting success. Patients progressing to injections generally had not responded to other medical therapies and thus were routinely weaned from neuromodulator medications following iSLN injection. Medications such as PPI or inhaled corticosteroids were not weaned. Those engaged with a speech–language pathologist were encouraged to continue cough retraining therapy during the period of active steroid effect, on the basis that behavioural consolidation during a window of reduced laryngeal hypersensitivity may enhance durable benefit.

### 3.3. Laryngoscopic Findings

Both groups underwent flexible high-definition laryngoscopy and stroboscopy on the day of the procedure. Among those with notable abnormalities, the most common finding was mild vocal fold oedema or erythema and paradoxical vocal fold movement. Vocal fold lesions were observed in some instances and one case of vocal fold paresis in the modified group. In the modified technique group, submucosal triamcinolone deposition in the region of the aryepiglottic fold and false fold was visible endoscopically at the time of injection as characteristic mucosal blanching and focal submucosal fullness. Very rarely a residual pocket of white triamcinolone was still visible at the 6-week mark post-injection. Stroboscopy was repeated at each follow-up visit as part of standard clinical practice; no cases of vocal fold stiffness attributable to steroid deposition were identified on vibratory assessment.

### 3.4. Procedural Characteristics

In the unmodified group, 17% (11/65) of patients achieved sufficient relief from a single unilateral injection and did not undergo a contralateral injection during the treatment period. The remaining 54 patients (83%) required bilateral treatment to target both iSLNs due to partial or no improvement from initial injection and the persistence of residual cough. In the unmodified group, patients who had an initial unilateral injection and experienced only partial improvement were subsequently offered contralateral injection at the next visit, progressing to bilateral coverage. In contrast, 76/77 (98.7%) of those in the modified technique group received bilateral iSLN injections as the default approach. To address the higher proportion of bilateral injections in the modified cohort, a bilateral-only sensitivity analysis was performed. The modified technique remained associated with a higher rate of patient-reported improvement compared to the unmodified technique (58/75, 77.3% vs. 24/54, 44.4%; Fisher’s exact *p* < 0.001) and greater median percentage cough improvement (75.0% (IQR 47.2–85.7) vs. 14.3% (IQR 0.0–57.1), *p* < 0.001).

The median number of iSLN block procedures per patient was two across both cohorts (mean injections; modified = 2.2 ± 2.9, unmodified = 1.9 ± 1.4; *p* = 0.56). One modified cohort refractory case during the observation period underwent multiple repeat injections (nine procedures over 3 years). The average interval between procedures (for those with >1) was approximately 8–9 weeks (*p* = 0.74). The decision to proceed with repeat injection was clinician-initiated within a shared decision-making framework. Patients were reviewed at approximately 6–8 weeks post-procedure; repeat injection was offered where partial benefit was observed and the patient wished to continue procedural management. No patients self-referred for repeat injection outside the standard follow-up pathway.

### 3.5. Adverse Events

Both techniques were associated with low complication rates, with no serious complications recorded in either cohort. In the unmodified group, three patients (4.6%) experienced minor adverse events; one vasovagal faint, one case of transient hoarseness lasting 1 week post-injection, and one patient with local ecchymosis at the injection site on the neck. In the modified group, five patients (6.5%) had minor events including two noting transient pre-syncope, one reporting self-limiting globus sensation, one with a transient increase in cough for 24 h post-procedure attributable to throat irritation, and one patient experiencing emesis shortly following injection while being monitored in the clinic. Of note, no cases of aspiration or persistent dysphagia were observed. Universally, there were no instances of prolonged laryngeal anaesthesia greater than expected with ropivacaine. The procedure was tolerated appropriately overall, and there were no procedure-related hospitalisations. There was no statistically significant difference between adverse events in either cohort (Fisher’s exact test, *p* = 0.72) (see Table 2).

### 3.6. Cough Improvement Outcomes

In the unmodified group, there was varied response. Whilst a subset achieved substantial relief, many had minimal change. Of the cohort, 47.7% reported symptomatic benefit, with a median reduction of 50.3% (IQR 25.0% to 75.0%). Contrastingly, patient-reported percentage improvement in cough at follow-up was significantly higher in the modified iSLN block cohort with a median improvement of 77.8% (IQR 61.3% to 86.6%) and 84.4% of patients responding to the intervention (Fisher’s exact test, *p* = 2.23 × 10^−5^) (see Table 3).

Response to the intervention can also be characterised via reported ≥50% symptom improvement. By that measure, 75.4% compared to 30.3% responded to the intervention in the modified and unmodified cohorts respectively (Fisher’s exact test, *p*= 1.53 × 10^−6^). Furthermore, complete or near-complete resolution (≥90% improvement) of cough was attained in 18.2% (n = 14) with the modified approach, versus only 6.2% (n = 4) with the unmodified approach (Fisher’s exact test, *p* = 0.041). Conversely, a lack of significant benefit (<10% improvement) was noted in 40% of the unmodified cohort, in comparison to only 13% of modified patients.

**Figure 1:**
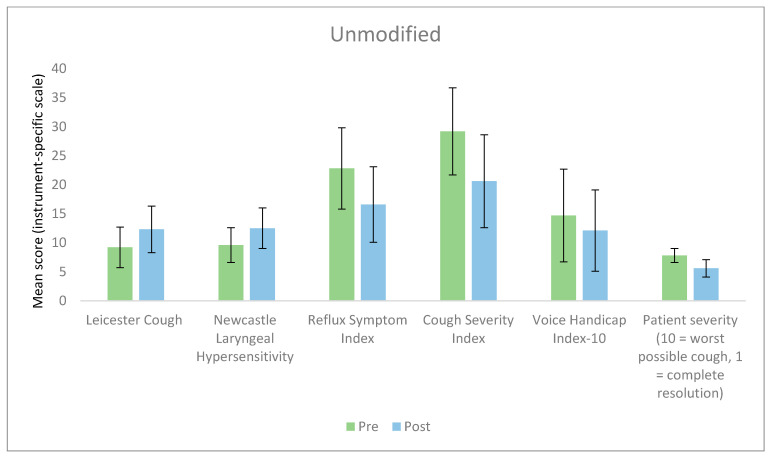
Mean (+/−SD) pre–post comparison of patient-reported outcome measures in the unmodified cohort.

**Figure 2:**
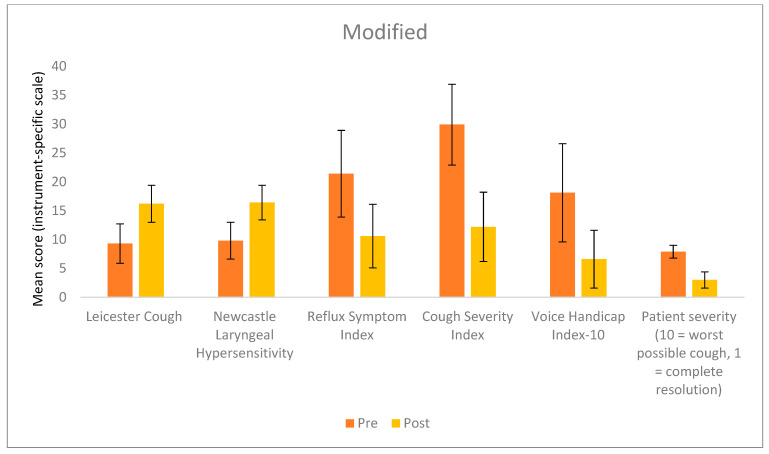
Mean (+/−SD) pre–post comparison of patient-reported outcome measures in the modified cohort.

These findings were supported by median improvements across outcome measures, with greater improvement in the modified cohort compared with the unmodified cohort for LCQ (8.3 vs. 1.2; Mann–Whitney U *p* = 4.12 × 10^−7^). Similar results stemmed from the CSI (22.0 vs. 3.0; *p* = 2.41 × 10^−6^), NLHQ (8.4 vs. 0.9; *p* = 8.61 × 10^−6^), RSI (11.0 vs. 4.0; *p* = 1.23 × 10^−4^), and VHI-10 (10.0 vs. 2.0; *p* = 1.62 × 10^−6^). Furthermore, patient-reported severity demonstrated greater improvements with the modified technique (6.0 vs. 1.0; *p* = 1.08 × 10^−6^) (see Table 4) (see Figure 1 and Figure 2).

## 4. Discussion

This retrospective comparative study found that a modified in-office iSLN block technique utilising nasoendoscopic visualisation and concurrent laryngeal evaluation is a valuable asset in treating refractory chronic cough. It was significantly effective in alleviating disease burden in those with chronic cough. This optimised method offers patients a probability of experiencing a significant degree of relief. Patients treated with the modified injection were more likely to experience substantial cough reduction and reported greater improvements on numerous validated cough outcome measures. When compared with the unmodified technique, this approach also proved its equality in terms of safety and tolerance, with no serious reported complications.

Our results expand on the prior evidence supporting iSLN blocks as an effective selection for refractory chronic cough. The initial study of Simpson et al. employing a combined corticosteroid and local anaesthetic injectable noted substantial improvement in CSI scores (mean; 26.8 pre-intervention to 14.6 post-intervention) [8]. Succeeding series have replicated clinically consequential improvements, including Dhillon et al.’s case series reporting significant CSI improvement [10]. Duffy et al. further suggested that response may not be uniform across all patients, identifying videostroboscopy vocal fold vibratory abnormalities as a potential marker of greater therapeutic effect [11]. The evidence for this intervention was reinforced by a placebo-controlled trial. Tipton et al. demonstrated via patient-reported and LCQ scores (treatment arm; 10.09 to 13.15) that iSLN block was associated with substantially higher improvement rate than saline placebo, including an absence of serious adverse events [12]. Bilateral injection strategies have accumulated a growing evidence base. A 163-patient series produced an average symptom reduction of 67.3%, with an average LCQ improvement of 4.11 [9]. Similarly, Bowen et al. reported bilateral concurrent iSLN injections as effective with potential to reduce a patient’s visit count whilst receiving the same intended benefit [13].

The modified approach described in this study may produce a superior outcome due to the culmination of several factors. Potentially, improved anatomic targeting via direct visualisation allowing real-time feedback that the treatment enters its desired target, as well as possible reduction in local mucosal inflammation reducing superficial laryngeal hypersensitivity. Comparatively, the landmark-guided unmodified technique may be less predictable due to pure reliance on surface anatomy and variation in tissue planes across individuals, potentially delivering the product incorrectly and leading to partial or absent sensory modulation. Also, concurrent endoscopic examination on multiple occasions may serve to enhance diagnosis.

Those considering management of cough, resistant to traditional medical therapies, should consider the adoption of bilateral iSLN injections including superficial laryngeal mucosa under endoscopic guidance to maximise patient benefit. The modified technique does require slightly more resources, including a flexible nasolaryngoscope and an assistant to hold the scope during the procedure. However, the ability to conduct the procedure in-office provides an accessible tool alongside medical and speech therapy [14]. Patients were followed up for recurrence of cough symptoms at follow-up visits. For the majority of participants, relief from symptomology was temporary in both cohorts, consistent with prior reports that the effect of an iSLN block may wane after several weeks [8,10]. Prior investigation has reported a mean duration of 7.6 weeks with bilateral blockade with a median of 4 weeks between symptom relief and subsequent injections [9]. Given the natural history of chronic cough, appropriate counselling should be provided to patients, informing them that injection procedures may need to be repeated, cough retraining therapy when the steroid is active being critical, and potentially combination with other therapies is advisable [15].

Regarding safety, whilst the absence of serious complications across 142 patients is reassuring, this cohort size is insufficient to exclude rare but clinically significant adverse events, and the favourable safety profile should not be interpreted as definitive. The thyrohyoid approach is performed in close proximity to the superior laryngeal artery and vein, and risks of inadvertent intravascular injection, cervical haematoma, and airway compromise, while not encountered in this series, remain relevant theoretical concerns—particularly should the technique be adopted more broadly outside highly specialised practice. The risk of bilateral sensory impairment with consequent reduction in protective laryngeal sensation and potential for aspiration is of particular relevance when bilateral blocks are performed and warrants careful patient selection and post-procedural monitoring. Aspiration prior to injection, appropriate patient positioning, a mandatory post-procedural observation period, and access to resuscitation equipment are recommended precautions. Larger prospective registries will be required to characterise the true rare-event risk profile of this technique.

The primary limitation of this study is that it was a retrospective analysis of prospectively collected data. Patients were not randomised to either technique, introducing the possibility of selection bias and temporal confounding. The asymmetry in mean follow-up duration between cohorts (1.9 years unmodified vs. 0.8 years modified), reflecting the more recent introduction of the modified technique, limits direct long-term comparison and may have influenced outcome ascertainment.

Concurrent neuromodulator use, proton pump inhibitor therapy, and ongoing speech–language pathology input were not controlled for and may have contributed independently to the observed improvements in patient-reported outcomes in both cohorts.

It is acknowledged that increasing familiarity with iSLN blockade over the study period may have influenced the threshold at which injections were offered, representing a potential source of temporal confounding.

The single-surgeon, single-centre nature of this study limits generalisability. The modified technique was developed and refined within a high-volume tertiary laryngology practice by a surgeon with extensive experience in flexible nasolaryngoscopy and in-office laryngeal injection. The technique is more technically demanding than the conventional approach, requiring simultaneous endoscopic visualisation, real-time interpretation of laryngeal landmarks, and percutaneous needle placement—skills that may not be readily transferable to lower-volume or general ENT settings without appropriate training and, where possible, supervised mentorship.

Prospective studies could be implemented to further support the findings of this study. Quantification of optimal dosing and whether the addition of agents to the injectable formulation may prolong effect would be worthwhile. Supplementary investigation could assess the ideal timing or frequency of injections and refine patient selection criteria.

## 5. Conclusions

As chronic cough management advances, injectables continue to emerge as an important therapeutic option to offer a transition between conservative therapy and more invasive measures. In this retrospective analysis of prospectively collected data, a modified iSLN block technique by using nasoendoscopic guidance and performing bilateral injections into the superficial laryngeal mucosa demonstrates superior outcomes for patients with recalcitrant chronic cough, without added risk. This technique utilises precision anatomical targeting of the iSLN and may impact on local inflammatory markers in the endolarynx. The result is a safe, in-office intervention that can significantly improve cough symptoms in appropriately selected patients. Adoption outside specialist laryngology practice should be approached with appropriate training, supervised mentorship, and an awareness of the potential risks inherent to the thyrohyoid approach.

## Figures and Tables

**Table 1:** Baseline Demographics and Clinical Characteristics.

	Unmodified (n = 65)	Modified (n = 77)
Age (median years)	60.8	63.8
Female	49 (75.4%)	57 (74.0%)
Smoking status—current	1 (1.5%)	5 (6.5%)
Smoking status—former	16 (20.0%)	18 (23.4%)
Smoking status—never	48 (73.8%)	54 (70.1%)
COPD	4 (6.2%)	12 (15.6%)
Asthma	17 (26.2%)	33 (42.9%)
GORD	35 (53.8%)	54 (70.1%)
ACEi use	3 (4.6%)	3 (3.9%)
LRTD	3 (4.6%)	14 (18.2%)
Allergic bronchial asthma	1 (1.5%)	3 (3.9%)
Allergic rhinitis	10 (15.4%)	20 (26.0%)
Post-nasal drip	17 (26.2%)	30 (39.0%)
Vagus nerve injury	0 (0%)	1 (1.3%)

**Table 2:** Procedural Characteristics and Adverse Events.

	Unmodified (n = 65)	Modified (n = 77)
Speech pathology (cough therapy)	53 (81.5%)	63 (81.8%)
Cough duration prior to SLN block (mean years)	10.0	8.8
Physical trigger point	7 (10.8%)	2 (2.6%)
Injection laterality—unilateral	11 (16.9%)	1 (1.3%)
Injection laterality—bilateral	54 (83.1%)	76 (98.7%)
Number of procedures (mean)	1.9	2.2
Interval between procedures (mean weeks)	9.0	8.2
Mean longitudinal follow-up (years)	1.9	0.8
Adverse effects	3 (4.6%)	5 (6.5%)

**Table 3:** Patient Self-Reported Improvement at Follow-Up.

Cohort	Improved—Yes (%)	Improved—No (%)	*p* Value (Between Groups)
Unmodified	31 (47.7%)	34 (52.3%)	*p* = 0.00002 (Fisher’s exact)
Modified	65 (84.4%)	12 (15.6%)	

**Table 4:** Pre- and Post-Injection Patient-Reported Outcome Measures.

Outcome Measure	Unmodified Pre (Mean ± SD)	Unmodified Post (Mean ± SD)	Within-Group *p* Value *	Modified Pre (Mean ± SD)	Modified Post (Mean ± SD)	Within-Group *p* Value *
Leicester Cough Questionnaire	9.2 ± 3.5	12.3 ± 4.0	0.08	9.3 ± 3.4	16.2 ± 3.2	<0.05
Newcastle Laryngeal Hypersensitivity Questionnaire	9.6 ± 3.0	12.5 ± 3.5	0.098	9.8 ± 3.2	16.4 ± 3.0	<0.001
Reflux Symptom Index	22.8 ± 7.0	16.6 ± 6.5	<0.05	21.4 ± 7.5	10.6 ± 5.5	<0.001
Cough Severity Index	29.2 ± 7.5	20.6 ± 8.0	<0.05	29.9 ± 7.0	12.2 ± 6.0	<0.001
Voice Handicap Index-10	14.7 ± 8.0	12.1 ± 7.0	0.12	18.1 ± 8.5	6.6 ± 5.0	<0.05
Patient-reported severity (1–10)	7.8 ± 1.2	5.6 ± 1.5	<0.05	7.9 ± 1.1	3.0 ± 1.4	<0.05

* Wilcoxon signed-rank test for within-group pre- to post-injection comparison.

## Data Availability

The data presented in this study are available on request from the corresponding author. The data are not publicly available due to patient privacy considerations.
